# Lymph Node Cellular and Viral Dynamics in Natural Hosts and Impact for HIV Cure Strategies

**DOI:** 10.3389/fimmu.2018.00780

**Published:** 2018-04-19

**Authors:** Nicolas Huot, Steven E. Bosinger, Mirko Paiardini, R. Keith Reeves, Michaela Müller-Trutwin

**Affiliations:** ^1^HIV Inflammation and Persistence Unit, Institut Pasteur, Paris, France; ^2^Vaccine Research Institute, Créteil, France; ^3^Yerkes National Primate Research Center, Emory University School of Medicine, Atlanta, GA, United States; ^4^Yerkes Nonhuman Primate Genomics Core, Yerkes National Primate Research Center, Atlanta, GA, United States; ^5^Center for Virology and Vaccine Research, Beth Israel Deaconess Medical Center (BIDMC), Harvard Medical School, Boston, MA, United States; ^6^Ragon Institute of MGH, MIT and Harvard, Cambridge, MA, United States

**Keywords:** HIV, SIV, natural hosts, lymph nodes, viral control, T cells, NK cells, inflammation

## Abstract

Combined antiretroviral therapies (cARTs) efficiently control HIV replication leading to undetectable viremia and drastic increases in lifespan of people living with HIV. However, cART does not cure HIV infection as virus persists in cellular and anatomical reservoirs, from which the virus generally rebounds soon after cART cessation. One major anatomical reservoir are lymph node (LN) follicles, where HIV persists through replication in follicular helper T cells and is also trapped by follicular dendritic cells. Natural hosts of SIV, such as African green monkeys and sooty mangabeys, generally do not progress to disease although displaying persistently high viremia. Strikingly, these hosts mount a strong control of viral replication in LN follicles shortly after peak viremia that lasts throughout infection. Herein, we discuss the potential interplay between viral control in LNs and the resolution of inflammation, which is characteristic for natural hosts. We furthermore detail the differences that exist between non-pathogenic SIV infection in natural hosts and pathogenic HIV/SIV infection in humans and macaques regarding virus target cells and replication dynamics in LNs. Several mechanisms have been proposed to be implicated in the strong control of viral replication in natural host’s LNs, such as NK cell-mediated control, that will be reviewed here, together with lessons and limitations of *in vivo* cell depletion studies that have been performed in natural hosts. Finally, we discuss the impact that these insights on viral dynamics and host responses in LNs of natural hosts have for the development of strategies toward HIV cure.

## Introduction

Combined antiretroviral therapy (cART) has transformed HIV infection from a lethal disease into a manageable chronic infection (1). Indeed, cART efficiently controls HIV replication leading to undetectable virus in blood and drastic increases in lifespan of people living with HIV (2). However, cART does not cure HIV infection as virus persists in cellular and anatomical reservoirs, from which the virus most often rapidly rebounds after cART interruption (3, 4). HIV probably rebounds from multiple sources (5). Virus-producing cells can be detected in SIVmac-infected macaques under suppressive cART in nearly every tissue, and in particular in the mucosal tissues and secondary lymphoid organs (6, 7). A major anatomical viral reservoir corresponds to lymph node (LN) B cell follicles, where HIV-1/SIVmac replication persists in follicular helper T cells (T_FH_) even in Elite controllers and cART-virologically suppressed individuals (8, 9). Surprisingly, T_FH_ cells expand during HIV-1 and SIVmac infections (10). Thus, lymphoid follicles have come to be considered as major sanctuaries for HIV/SIV (9). In parallel, HIV-1 and SIVmac might also persist in some CD4^+^ T cells within the T zone of LN during cART (11). In this review, we will focus on the viral and host dynamics in LNs of natural hosts and discuss similarities and key differences with regard to HIV and SIVmac infections.

## Primary Characteristics of Non-Pathogenic SIV Infection in Natural Hosts

Natural hosts of SIV, such as African green monkeys (AGMs) (*Chlorocebus aethiops*), sooty mangabeys (SMs) (*Cercocebus atys*), and mandrills (*Papio sphinx*), generally do not progress to disease despite displaying persistently high viremia (12–16). The vast majority of the studies carried out on SIV infections in natural hosts have been performed using two species, SMs and AGMs (17). The comparison of the clinical, virological, and immunological parameters of infection in these species with that of HIV/SIVmac infections allowed advances in knowledge on the mechanisms linked to protection against AIDS. In particular, natural hosts rapidly resolve inflammation induced by SIV infection, and unlike pathogenic lentivirus infections do not develop chronic immune activation (see chapter below).

An important aspect of SIV infection in natural hosts is also their ability to preserve the function and structure of their tertiary and secondary lymphoid organs throughout the infection. Indeed, natural hosts avoid the widespread damage to the mucosal immune architecture that is observed in pathogenic infections (Table 1). While acute SIV infection leads to a rapid, near-complete loss of CD4^+^ T cells in the intestine in both natural hosts and macaques, mucosal CD4^+^ T cells partially recover in natural hosts, even if not to baseline levels (18–21). Furthermore, cART administration to SIV^+^ SM induces a rapid and substantial recovery of mucosal CD4^+^ T cells that is not typically observed in HIV infection (22). Moreover, despite high viremia and high-level replication in the gut (23), natural hosts, in stark contrast to non-natural hosts, preserve intestinal Th17 cells (24, 25), retain the structural integrity of the mucosal barrier (26), and do not exhibit leakage of mucosal lumenal microbiota (i.e., microbial translocation) into systemic circulation (27–29). With regard to LN during SIV infection in natural hosts, there is generally no sign of lymphadenopathia nor fibrosis and LN display a normal follicular dendritic cell (FDC) network (12, 30, 31) (Table 1). Another characteristic of natural hosts is the relatively low infection of central memory T cells (see below) (32). Natural hosts thus seem to have developed ways to protect the sites of education and memory of immune responses.

**Table 1:** Major similarities and differences between HIV/SIVmac infections and SIV infections in natural hosts at the level of lymph nodes (LNs).

LNs		Natural host (African green monkeys, sooty mangabeys)	Non-natural host (human/macaque)	Reference
Viral replication in LN (17, 12, 34)	Acute phase	High	High	(17, 33, 34)
	Chronic phase	**Low**	**High**	

Inflammation	Acute phase	**Rapid**	**Strong**	(35–39)
	Chronic phase	**No**	**Yes**	
	IFN-a	High in acute infection	High in acute infection	
	Interferon-stimulated gene	High in acute infection	**High in acute and chronic infection**	
	TGF-β and collagen deposition	**No**	**Yes**	

LN architecture	Lymphadenopathia	**No**	**Yes**	(12, 30, 40)
	Follicular dendritic cell network	**Preserved**	**Lost**	
	Fibrosis	**No**	**Yes**	

Location of SIV-infected cells	T cell zone	Yes	Yes	(11, 12, 41, 42)
	B cell follicles	**Rare/absent**	**Yes**	
	Virus trapping	**Rare/absent**	**Yes**	

SIV-infected cells	CD4^+^ T_CM_	**Low**	**High**	(43–47)
	T_CM_ PD-1^+^CTLA4^+^	nd	Yes	
	T_FH_	**Rare/absent**	**High**	
	Plasmacytoid dendritic cell	Yes	Yes	
	macrophage	Yes	Yes	

Antiviral immune responses	HIV/SIV-specific T cell responses	Weak	Variable 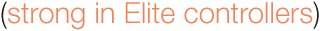	(17, 48–50)
	Follicular CD8^+^ T cells	nd	Yes (rare)	
	Follicular NK cells	**Yes**	Yes (rare)	
	bNAb	nd	Yes (rare)	

*The green and red colors highlight, respectively, major differences between SIV infection in natural hosts and HIV/SIV infections in non-natural hosts*.

## Viral Dynamics in LNs during SIV Infection in Natural Hosts

Studies in SIVmac infection have shown that the viral seeding of LN occurs rapidly and progressively. One to three days after infection, some replicative viruses could already be detected in the draining LN and even in systemic LN (51). Of note, during the eclipse phase until peak viremia, productively infected cells are found essentially in the extra-follicular zone of LN (41). Only in later phases of primary infection, and in particular during chronic infection, viral RNA is found inside B cell follicles, where it replicates within T_FH_ cells (45). In addition, virus is trapped within the follicles by FDCs where it remains infectious for 9 months or more (30, 52, 53). The mechanism driving this shift from the T cell zone to the B cell follicles is incompletely understood.

During chronic HIV/SIVmac infections, virus replication in LN exceeds the levels in blood by several orders of magnitude. In ART-naive SIVmac infection, LN are estimated to support ~50% of viral burden, and be reduced to ~1% in the context of suppressive ART, with the remainder supported by mucosal tissue (6). In one SIVmac-infected macaque, the frequency of infected cells in LN was evaluated and appeared to be as high or slightly higher than in the gut (mean frequency ~8.7 × 10^5^ vRNA^+^ cells/g in LN and ~5.6 × 10^5^ vRNA^+^ cells/g in the gut) (6). ART administered for >20 weeks decreased the mean frequency of vRNA in LN by approximately 2 log_10_ in SIVmac_251_-infected rhesus macaques (6).

The reason of the preservation of the normal architecture of LN in natural hosts might be associated with the significantly lower levels of viral replication in this tissue. Strikingly indeed, AGM and SM mount a strong viral control in LN shortly after peak viremia, which lasts throughout infection (12, 23, 43, 54–56). Thus, while during the first 2 weeks post-infection (p.i.), the number of productively infected cells as well as the copy numbers of cell-associated viral DNA and RNA are similar between SIV infection in natural hosts and macaques, major differences are observed after the viremia peak between natural and non-natural hosts (12, 49, 56). Thus in natural hosts, viral replication levels decrease drastically in LNs after peak viremia, whereas in pathogenic infections, after a moderate decrease, a relatively strong viral replication generally persists throughout the infection in absence of cART, leading to a difference of 2–3 log in the cell-associated viral RNA in LN during chronic infection between macaques and natural hosts. Viral RNA-producing cells as well as cell-associated viral RNA sometimes become even undetectable in LN of AGM, despite continuous high-level plasma viremia (12, 14, 33).

**Figure 1:**
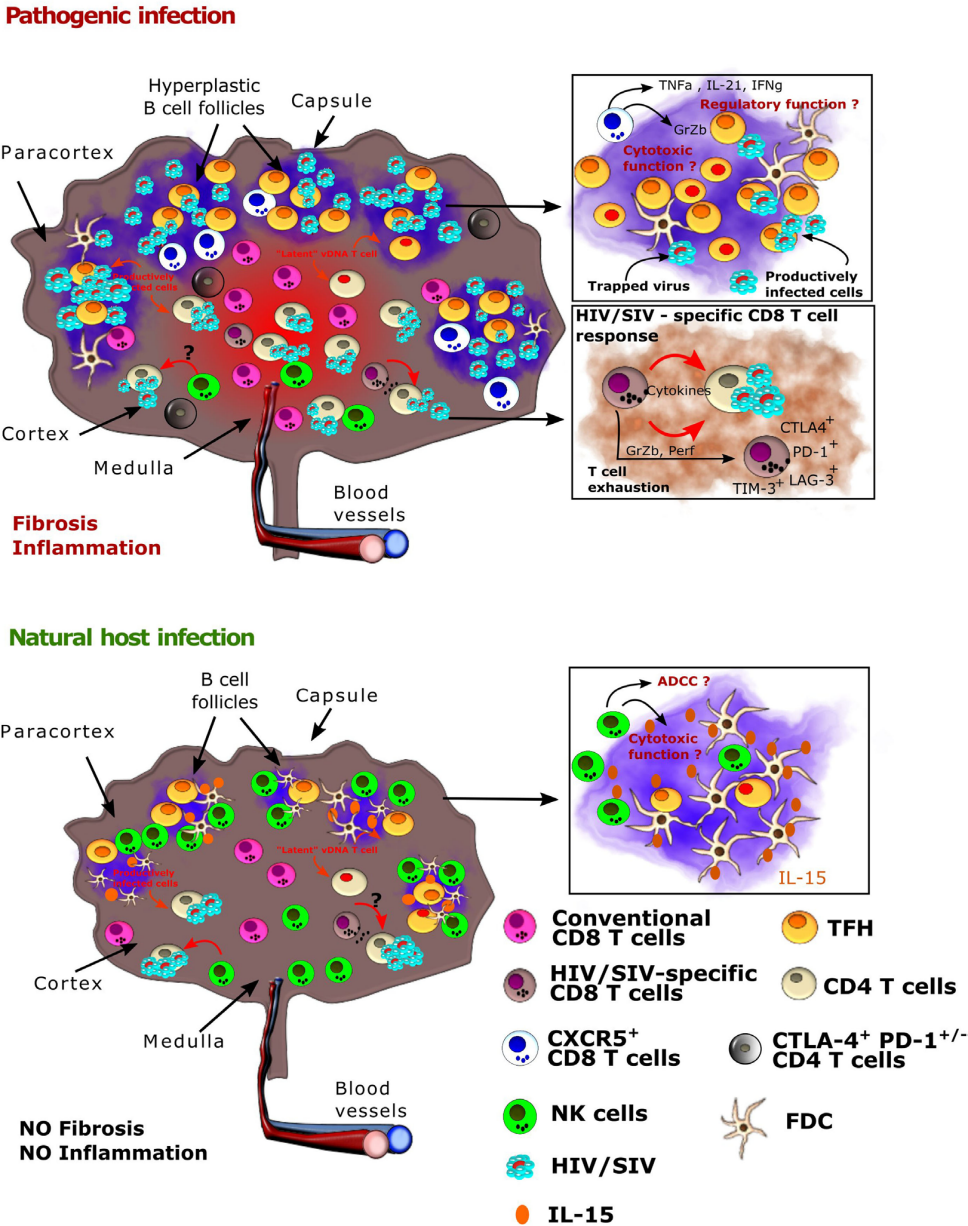
Viral and host immune cell dynamics in lymph nodes (LNs) from natural hosts versus HIV-1/SIVmac infections. Schematic representation of a LN after HIV or SIV infection in pathogenic models (human, macaques, top) and natural hosts [African green monkey (AGM), sooty mangabey, bottom]. (*Top*) HIV-1 and SIVmac infection in, respectively, humans and macaques result in the formation of hyperplastic germinal centers in LNs with massive B cell proliferation. T_FH_ cells also expand during HIV-1 and SIVmac infections. Inflammation is uncontrolled and leads to collagen deposition and fibrosis. The follicular dendritic cell (FDC) network is disrupted on the long term. HIV-1 and SIVmac replicates in combined antiretroviral therapy (cART)-naïve individuals and animals in both T and B cell zones, but the viral burden is highest in the B cell zones (follicles). In the follicles, virus replication is concentrated within follicular helper T cells (T_FH_). Virus is also trapped by FDC and remains infectious. On cART, virus persists mostly in T_FH_ cells in the follicles, where it is often outreach of conventional CD8^+^ T cells and of optimal drug concentrations, as well as in CTLA4^+^CD4^+^ T cells within the T zone. The latter cells have a capacity for long survival. NK cells and conventional HIV/SIV-specific CD8^+^T cells are often expressing immune checkpoint inhibitors. The presence of CXRC5^+^CD8^+^T lymphocytes has been described, but their role needs to be further studied. (*Bottom*) In natural hosts, virus replication is strongly controlled during the chronic phase of infection. Most follicles are exempt of virus. Conventional SIV-specific CD8^+^ T cell responses are weak. NK cells play a major role in the control of viral replication in AGM LNs. Both the IFN-α and NK cell responses appear earlier than in SIVmac-infected macaques. NK cells accumulate in follicles in SIVagm-infected AGMs, which might be a direct consequence of a high production of IL-15 in the follicles. NK cell migration into B cell follicles in response to SIVagm infection is associated with the acquisition of CXCR5. CXCR5^+^ NK cells express high levels of Fcγ receptors and of CD107a, which raises the question if they have the capacity to control SIVagm replication through antibody*-*dependent and/or -independent cellular cytotoxicity.

The anatomical distribution of virus replication in chronic infection is also very different between non-pathogenic and pathogenic infection. Indeed, in natural hosts, most virus is detected in the T cell zone, even if at extremely low levels, while in pathogenic HIV/SIV infection, most virus is present in follicles (Figure 1). Strikingly, in natural hosts, such as AGM and to a lesser extent in SM, viral RNA is generally absent in follicles. This is not a matter of the virus, as SIVsm and SIVagm infections of macaques lead to high SIV levels in follicles (57, 58). Natural hosts are thus characterized by a limited or absent replication in T_FH_ cells and frequent lack of FDC deposition of virus (35, 59). Understanding the underlying mechanisms of the strong viral control in LN in natural hosts might yield clues helpful for the development of strategies aiming the elimination of HIV reservoirs in follicles.

## Regulation of Inflammation in LNs and Impact on SIV Infection in Natural Hosts

The deleterious impact of unabated inflammation in HIV infection has been well documented (35). This immune activation is positively correlated with HIV-1/SIVmac replication in both ART-naïve and ART-treated settings (60, 61). Among the myriad of detrimental manifestations due to the persisting inflammation that have been reported, a handful could be particularly influential in maintaining viral burden, namely: (i) recruitment of target cells, (ii) impairment/exhaustion of adaptive immunity, and (iii) the disruption of lymphoid structures. In this section, we will review existing data on inflammatory pathways differing significantly between natural hosts versus pathogenic SIV infection. These data will be reviewed in the context of the natural host’s low-to-absent SIV burden in LN follicles. Hypotheses concerning the effect of non-natural inflammation in supporting LN SIV replication will be presented.

A longstanding observation in natural hosts is that they are devoid of the pan-lymphocyte activation and chronic inflammation seen in pathogenic HIV/SIV disease (62–64). The molecular and immunological distinctions of these species have been extensively characterized [reviewed in Ref. (35, 44, 65)]. Although natural hosts exhibit levels of immune activation similar to baseline during chronic infection, detailed longitudinal studies have demonstrated that rapid, early immune activation is evident, including elevated levels of IFN-a, CD8^+^Ki67^+^ T cells and PD1 expression in LN (63, 66, 67). The most striking confirmation is the massive upregulation of interferon-stimulated gene (ISG) expressions during acute infection in natural hosts (68, 69) in blood, LN, and gut. These ISGs include many antiviral restriction factors, such as MX2 and Tetherin. Of note, the upregulation of ISGs occurs very early, starting from days 1 or 2 p.i. in AGM, concomitantly with a very early transient increase in IFN-α (68, 70). By contrast, during SIVmac infection in macaques, it was reported that the expression of those ISGs encoding antiviral restriction factors was delayed and not upregulated before peak viral replication on day 10 (71). Thus, natural hosts seem to develop a more rapid antiviral innate response to SIV compared to non-natural hosts (66, 68, 70, 71). Subsequently, natural hosts rapidly resolve total ISG expression to baseline before the transition to chronic infection despite prevalent viremia. This downregulation of ISG expression in natural hosts is in stark contrast to HIV/SIVmac infections, in which ISG expression remains elevated indefinitely (72).

The observation that natural hosts resolve IFN-I related responses prompted a series of comparative studies into plasmacytoid dendritic cells (pDCs). pDC trafficking to LN has been reported for both natural and non-natural hosts. A peak of pDC accumulation in LN is observed approximately 7–14 days after SIV infection in macaques, SM, and AGMs concomitant with robust IFN-α and IFN-β *in situ* production by pDC in LN (66, 73–76). The trafficking of pDC to tissues during SIV infection differs in several aspects between natural hosts and non-natural infections: (i) in AGM, an early first peak of pDC in LN is observed around days 1–3 p.i. (66); (ii) pDC accumulate in the rectal mucosa in infected humans and macaques, but not in SM, which has been attributed to heightened levels of α4β7 in SIVmac infection (77, 78), and (iii) pDC in LN during acute SIVmac infection are prone to apoptosis, while for natural hosts this is not known (39, 73). Both SM and AGM were demonstrated to retain intact sensing and IFN-α production in pDC in response to their native SIV (68, 79–81). Of note, pDC from AGM sense more efficiently SIVagm than SIVmac or HIV-1 viruses (81). Studies in natural hosts have revealed that SIV infection alters the capacity of viral sensing in cells other than pDC, which then can also produce IFN-I during acute infection (80). The contribution of pDC to IFN responses during chronic SIV infection remains unresolved, while some reports have not detected IFN-I in pDC during chronic infection (74), we have observed IFN-α transcripts in LN pDC as far out as 18 months post-infection (Bosinger, unpublished observations).

The consequences of unabated IFN production on immune function and viral reservoirs in HIV infection are under intense study. IFN-induced responses are clearly critical for the control of SIV in LN during acute infection, as antagonism of the IFN-α receptors (IFNAR) from before infection to early time points p.i. in macaques caused elevated levels of LN-associated SIV and plasma viremia (82).

The effects of IFN during chronic HIV infection are less clear. Mouse models have shown that persistent TLR and IFN signaling causes damage to the lymphoid structures (83). Several studies have demonstrated that irreversible fibrosis is evident in the LNs of SIV-infected macaques, but, interestingly, is absent in natural host infection (31, 84). The fibrosis in chronic HIV/SIV infection might be linked to persistent IFN-related inflammation, TGF-β produced by regulatory T cells (Treg) leading to collagen deposition, and/or other yet unknown factors (84). Disruption of IFN-I signaling in chronic infection appears to have indeed a beneficial effect on host immunity in certain settings. In the mouse model of lymphocytic choriomeningitis clone 13 infection, blockade of IFN-β signaling in chronic infection enabled spontaneous clearance of the virus (85–87). In a remarkable set of independent studies using ART-suppressed, HIV-infected humanized mice, disruption of IFNAR signaling reduced latent HIV levels and ameliorated systemic immune activation (88, 89). In both the LCMV and hu-mouse HIV datasets, IFN-blockade reduced expression of co-inhibitory molecules on CD8^+^ T cells and improved cellular antiviral responses; thus, the mechanism of action was presumed to be alleviation of IFN-mediated exhaustion of T cell responses. These studies provide some rationale for IFN blockade to be applied as a therapy to lower the reservoir, but this hypothesis would first need validation of efficacy and safety in pre-clinical studies. Taken together, the observations that (i) SIV natural host species avoid long-term ISG expression and (ii) *in vivo* antagonism of type I IFN signaling can improve antiviral immunity and reduce reservoir levels in the hu-mouse model suggest that the overall contribution of IFN in chronic HIV/SIV infection is harmful by maintaining high levels of immune activation and contributing to immune dysfunction. However, exogenous administration of IFN-α to ART-suppressed, HIV-infected patients have shown in some cases clinical benefit in terms of reduced levels of cell-associated HIV DNA (90–92). Thus, the contribution of IFN-α to chronic inflammation and viral persistence during ART-treated HIV/SIV infections is still unclear. Injection of exogenous IFN-α into SIV-infected AGM and SM have not been able to reproduce the phenotype of widespread immune activation observed in non-natural hosts (70, 93). However, the injections of exogenous recombinant IFN-α induced a rapid state of tolerance *in vivo* to this molecule. It is not excluded that one might need to treat for long periods of time with intermediate breaks to see an effect on chronic inflammation. The other possible explanation is that IFN-I levels are not different between pathogenic and non-pathogenic infections and/or that IFN-I are not the major culprits of the persistent ISG expression (68, 70). Other factors, such as IFN-γ, might contribute to ISG upregulation (68, 94). Collectively these comparative studies in distinct models indicate that IFN-I signaling is (i) beneficial during acute infection, (ii) a major contributor to early immune activation, (iii) alone insufficient to cause chronic immune activation, and (iv) its impact is highly context dependent.

Several other factors have been put forward to explain the absence of chronic inflammation in natural hosts. For example, by sequencing for the first time the genome of the SM, a mutation was uncovered in the gene encoding TLR4, the primary receptor for LPS, that yields a truncated protein and attenuated signaling (95). Intriguingly, this mutation was also observed in the *TLR4* gene of the two other natural host species (AGM, mandrills) (95). This mutation might contribute to a lower monocyte/macrophage activation in natural hosts.

The maintenance of viral replication in LNs could impact systemic inflammation, due to the sheer immune “traffic” and recirculatory nature of immune cells. From this point of view, the fact that natural hosts strongly control viral replication in LN might contribute to their capacity to resolve inflammation. In this light, understanding the mechanisms by which HIV/SIV replication could be controlled in the LN is likely to be critical not only for viral eradication strategies but also for therapies aiming at reversing immune activation.

## Target Cells for SIV in LNs from Natural Hosts as Compared to Pathogenic HIV and SIVmac Infections

Reducing the persistent HIV/SIV reservoir remains an essential milestone for the achievement of a functional cure for HIV-1 infection; however, this goal has been significantly hindered by poor means for identification of the CD4^+^ T cell subsets that harbor replication-competent virus, as well as by the anatomic location of these cells in sanctuaries for HIV. Several key differences in the nature of cells targeted by SIV in natural versus non-natural hosts have been identified, raising the fascinating hypothesis that the type of infected CD4^+^ T cells, even more than the quantity, could contribute to the different capacity to control immune activation and disease progression between the two hosts.

### Central Memory CD4^+^ T Cells (TCM)

*In vivo* and *in vitro* comparative studies showed that the frequency of SIV-infected TCM in SM is significantly lower as compared to both CD4^+^ T effector memory cells of SM and CD4^+^ TCM of macaques in both blood and LN (32, 59). Thus, SM are partially protecting the important CD4^+^ TCM cell subset from SIV infection. In line with this relative preservation from viral infection, CD4^+^ TCM cells are more preserved in SIV-infected SM compared to SIV-infected rhesus macaques (46). CD4^+^ TCM cells are long-lived, self-renewing cells able to replenish the pool of non-self-renewing, shorter lived CD4^+^ effector memory cells, thus their maintenance is key for the homeostasis of the overall CD4 T cell compartment and immune memory. Remarkably, a low contribution of infected CD4^+^ TCM to the overall viral reservoir has similarly been described in (i) long-term non-progressors with protective HLA alleles (96); (ii) viremic non progressors, i.e., rare HIV-infected individuals who maintain high CD4^+^ T cell levels despite uncontrolled viremia (97); and (iii) post-treatment controllers, i.e., patients with a durable control of viremia after ART-interruption (98). With a distinct strategy, AGMs have also evolved to protect memory CD4^+^ T cells from viral infection. Indeed, CD4 molecules get downregulated from the surface of the CD4^+^ T cells when the latter get activated. Of note, these cells maintain their T helper functional activity (99).

The mechanisms of TCM protection are not clear. It has been suggested that CCR5 plays a role. Thus, CD4^+^ T cells from natural hosts express less CCR5 in blood, LN, and mucosae compared to humans and macaques (100, 101). It also has been shown that *in vitro* stimulation of SM CD4^+^ T cells, particularly the TCM, fail to upregulate CCR5 (32). CD4^+^ TCM cells expressing low levels of cell-surface CCR5 are less susceptible to SIV infection when compared to TCM of macaques both *in vivo* and *in vitro* (46, 102). However, SIV from natural hosts can also efficiently use other coreceptors than CCR5 to infect primary CD4^+^ T cells and other factors might as well be implicated in the relative preservation of TCM to infection in natural hosts (103). LN comprises a higher fraction of TCM compared to mucosal tissues, the latter containing higher proportions of effector cells in mammals (104). Thus it is possible that in natural hosts, the lower ratio of TCM infection is related to the control of viral replication in LN, whereas the predominant virus replication in the gut would explain why most virus infects CD4^+^ effector T cells in natural hosts. Altogether, the viral tissue distribution could thus in part also explain the lower frequency of infection rate in TCM compared to CD4^+^ effector T cells in natural hosts.

### Follicular Helper T Cells (T_FH_)

T_FH_ correspond to a subpopulation of memory CD4^+^ T cells expressing high levels of CXCR5 and PD-1 residing within the follicles of secondary lymphoid organs. They impact the activation, differentiation and survival of B cells. Several studies explored the frequencies, function, and infection rate of T_FH_ cells in HIV-infected humans or SIV-infected macaques. They revealed that T_FH_ cells are infected at high frequencies in chronic infection. Despite the high rate of HIV/SIVmac replication in T_FH_ cells, these cells expand during HIV and SIVmac infections (45, 59, 105, 106). More recently, it was shown that T_FH_ cells constitute an important source of persistent replication-competent virus in ART-treated, aviremic individuals (8). By contrast, a low infection rate of T_FH_ cells has been described during non-pathogenic infection of SM (59) and AGM (49), where follicles often remain virus free. LN T_FH_ cells showed lower levels of Ki-67 expression than non-T_FH_ memory CD4^+^ T cells and fewer of the T_FH_ cells expressed CCR5, but this was similar between macaques and natural hosts (59). Phenotypic studies on T_FH_ cells in natural hosts are though limited so far and whether T_FH_ cells in LN expand differently during SIV infection in natural hosts needs to be further investigated.

### CD4^+^PD-1^+^CTLA-4^+^ T Cells

The contribution of T_FH_ cells to the persistent reservoir progressively decreases with increased length of cART (8, 107), suggesting that other cell subsets, apart from T_FH_ cells, can contribute to the magnitude of the pool of latently infected cells. In a recent study, it was found that PD-1^+^ cells, the subset that contributes most to T_FH_ cells, were indeed the dominant contributors to the viral DNA pool in the B cell follicles in the LN in ART-treated SIV-infected macaques; however, CTLA-4^+^PD-1^−^ memory CD4^+^ T-cells, a subset comprised predominantly of Tregs, were identified as a previously unrecognized component of the SIV reservoir (11). These cells are significantly enriched in SIV DNA in multiple tissue compartments, including the blood, LN, spleen, and gut and have been shown to harbor replication-competent and infectious virus (11). CTLA-4^+^PD-1^−^ cells localized in the extra-follicular zones of the LN in ART-treated SIV-infected macaques and HIV-infected humans. Therefore, in addition to PD-1^+^ T_FH_ cells, HIV-1 and SIVmac are able to establish and maintain viral persistence through the specific targeting of another CD4^+^ T cell subset, CTLA-4^+^PD-1^−^ cells. These cells seem to have long living capacities (11). Further studies are needed to determine if the rare SIV-producing cells in the T zone of natural host’s LNs correspond, at least partially, to these CTLA-4^+^PD-1^−^ cells.

### Plasmacytoid Dendritic Cell

Unlike humans’ and macaques’ pDC, pDC from natural hosts display substantially lower CD4^+^ and CCR5^+^ surface expression (80). The lowered SIV receptor/coreceptor expression however does not affect the ability of SIVagm to infect pDCs. Indeed, high rates of pDC infection were detected in the spleen of AGM, to a similar high rate as pDC infection by HIV in cART-naïve humans (81).

## Potential Immune-Mediated Mechanisms for Viral Control in LN: The Role OF CD8^+^ T and NK Cells

There are several clear lines of evidence that CD8^+^ T cells play an important role for the overall control of HIV-1/SIVmac replication (108, 109). Some of the most convincing evidences have been obtained in macaques and include a temporal correlation between the rise of SIV-specific CD8^+^ T cells and post-peak viremia decline, as well as the increase of viremia after *in vivo* depletion of CD8^+^ cells (110). Of note, most *in vivo* depletion studies used monoclonal antibodies that did not discriminate between CD8^+^ T and NK cells, and thus in some of these studies, the contribution of NK cells remained undetermined. Nonetheless, the role of CD8^+^ T cells in viral control is undeniable and is evident in HIV controllers and rhesus macaques with specific MHC alleles (111, 112).

CD8^+^ T cells in LN are generally located in the T cell zones. Early studies have revealed massive infiltrations of activated CD8^+^ T cells into B cell follicles in progressors, but this could be due to the disruption of the FDC network in late stage disease (113–116). Nevertheless, the magnitude of fully cytolytic CD8^+^ T cells was significantly higher in LN compared to blood (117), and HIV-1-specific CD8^+^ T cells are preferentially located in LN compared to blood, including a subset of responses that is present solely in secondary lymphoid organs (118). This preferential location of HIV-1-specific CD8^+^ T cells in the LN was also observed in chronically infected individuals on cART (118). These migrating CD8^+^ T cells localize to the extra-follicular zones of the LNs, where most of endogenous HIV-1-specific CTL were also observed, far from sites of virus replication inside the follicles (117). After *in vivo* depletion of CD8^+^ cells in SIVmac-infected macaques, the frequency of SIV-infected cells in extra-follicular regions increased and reached levels similar to that in B cell follicles (9) confirming that CD8^+^ T cells exert control of viral replication predominantly in the T cell zones. Until recently, it was considered that CD8^+^ T cells generally do not migrate into the B cell follicles and it was further suggested that antiretroviral drugs inefficiently diffuse into or are unequally distributed within LN (84), collectively making follicles a prime sanctuary for HIV/SIV replication. Nonetheless, a small proportion of CD8^+^ T cells expressing CXCR5^+^ has been recently described in both SIVmac and HIV-1 infections (119–121). The levels of these CXCR5^+^CD8^+^ T cells in LN were higher in HIV-infected individuals compared to healthy donors, and they were detected in close proximity to viral RNA^+^ cells, probably starting from primary infection on (119, 122). The frequency of SIV-specific CXCR5^+^CD8^+^ T cells correlated negatively with that of SIV infection in T_FH_ cells and viremia, suggesting a role of CXCR5^+^CD8^+^ T cells in viral control (50). However, other studies highlighted a regulatory phenotype of CXCR5^+^CD8^+^ T cells with poor capacity of viral control which could further impair germinal center function in HIV infection (120, 123). Unfortunately, little is known about these recently described follicular CD8^+^ T cells, and whether the contrasting results are due to the presence of distinct CXCR5^+^CD8^+^ T cell subsets, differences in the infection models studied or other yet unknown factors.

In natural hosts, the contribution of CD8^+^ T cells to controlling SIV replication may be comparatively small. Indeed, although SIV-specific CD8^+^ T cell responses were observed for SIV-infected SM and AGM, their magnitude and breadth were similar or even lower than those generally observed in HIV-1 and SIVmac progressive infections both in blood and LN (124–127). However, it has been suggested that these responses appear temporally earlier in LN of natural hosts compared to pathogenic species and that this confers an advantage (56). Of note, these cells do not seem to migrate into follicles. CD8^+^ T cells from natural hosts were indeed found to be exclusively located in the T cell zones both in non-infected and SIV-infected animals (12). In line with this, CD8^+^ T cells in LN from AGM do not upregulate CXCR5 in response to SIV infection (49). To further address the question of the role(s) played by CD8^+^ T cells during natural host’s SIV infection, *in vivo* cell depletion experiments have been conducted. Administration of anti-CD8^+^ and anti-CD20^+^ antibodies during the first 2 weeks of SIVagm_ver90_ infection in pig-tailed macaques (pathogenic infection) and AGM (non-pathogenic infection), led to dramatically different results in the two species (128). In pig-tailed macaques, a one-log increase in peak viremia and four-log increase in set-point viremia were observed following antibody administrations. Moreover, these animals rapidly progressed toward disease and displayed CMV reactivation. By strong contrast, in AGM, depletion of CD8^+^ and CD20^+^ cells did not modify peak viremia and the animals displayed only a minor delay in post-peak viremia decline compared to control animals, and all animals remained clinically healthy (128). In another study, treatment of SIVsm-infected SM using a CD8α-specific Ab (OKT8F) led to a profound depletion of CD8^+^ cells in both blood and tissues such as LN, but only minor changes in plasma viremia (129). Similar results were also observed in AGMs in which CD8^+^ cell depletion during the acute phase led only to a delay of 5–10 days in the post-peak viral decline (130). By contrast, virtually all CD8^+^ *in vivo* depletion studies conducted in non-natural host models during acute or chronic SIV infection have reported significant increases in viral loads and rapid disease progression (110, 131–133). Altogether, these data highlight that while CTL responses can play a large role in HIV controllers, they may contribute only modestly to the control of viral replication in LN in natural hosts. Thus, while CD8^+^ T cells might still be involved to some extent in the control of viral replication in the T cell zone, they most likely do not represent the major cellular component of viral control in LN follicles during SIV infection in natural hosts.

As an alternative to CD8^+^ T cells, multiple lines of evidence pointed toward a role of NK cells in the control of SIV replication in LN of natural hosts. Upon SIV infection, AGM temporarily display high levels of IFN-α and IL-15 in the plasma (70). These cytokines are known to activate NK cells and enhance their cytotoxic profile (134, 135). Plasma IFN-α levels correlated indeed with activation and cytotoxic activity (CD107a) of NK cells and plasma IL-15 with the proliferation (Ki-67) of NK cells in LN during acute SIVagm infection (70). During the acute phase of SIVagm infection, CD107a^+^ NK cells increased to higher levels in LN than in blood (70). Studies in SM demonstrated a more rapid activation of NK cells compared to macaques (136, 137). These previous studies raised the hypothesis that NK cells may play a role in LN viral control in natural hosts. It was subsequently shown that upon SIVagm infection, NK cells change their distribution within LN and migrate into follicles, where they accumulate (49). The increase of NK cell numbers in follicles was associated with a high production of IL-15 within follicles, presented in membrane-bound form by FDC and antigen-presenting-like cells (49). By contrast, the number of functionally competent NK cells in LN decrease in macaques in response to SIV infection (49, 138). The pattern of LN homing receptors (CX3CR1, CD62L, CXCR3, CCR7) were similar on NK cells from SIV-infected AGM and MAC and do not explain the higher levels of NK cells in LN of AGM as compared to MAC (49). It is more likely that in SIVagm infection, the IL-15 in the follicles enhances the survival of NK cells. Interestingly, SIVagm-infected AGM showed high levels of CXCR5^+^ NK cells in LN (49). This suggests that migration of NK cells into AGM follicles was CXCR5-mediated. The presence of CXCR5^+^ NK cells was observed in secondary lymphoid organs (LN, spleen), but not in blood or gut of SIV-infected AGM. Thus, the CXCR5 expression on NK cells during SIVagm infection was tissue-specific. Of note, this enrichment of CXCR5^+^ NK cells in secondary lymphoid organs was not observed in SIVmac-infected macaques. Strikingly, IL-15-mediated depletion of NK cells in chronic SIVagm infection led to high viral replication in the follicles as well as in the T zones (49). These results indicate that T_FH_ cells are not resistant to SIV infection in AGM and clearly reveal a crucial role for NK cells in the viral control within LN of a natural host.

## Concluding Remarks

Herein, we summarize current knowledge on differences in LN of non-natural versus natural hosts. The remarkable control and clearance of virus from lymphoid follicles in natural hosts is associated with multiple differences compared to pathogenic infection: (1) LN architecture is preserved; (2) inflammation is controlled; (3) FDC network is maintained intact; (4) rapid mobilization of innate antiviral responses; (5) viral replication is strongly controlled; (6) T_FH_ are particularly spared from virus; (7) NK cells migrate into follicles; and (8) high IL-15 production within follicles (Table 1). Collectively, natural hosts have developed mechanisms of protection for the most vulnerable lymphoid CD4^+^ T cell subsets: CD4^+^ TCM, T_FH_ cells in LN, and Th17 cells in gut (35, 139). Better preservation of these cells likely influences the preservation of intact lymphoid structures, immune competencies, and immune memory (49, 55). As a control model for lentivirus infections, we must ask how we might exploit the knowledge garnered from natural host research. Given the IL-15-dependent accumulation of NK cells (and potentially CD8^+^ T cells) in natural hosts into follicles, this could be envisioned therapeutically to recapitulate virus clearance in pathogenic hosts and HIV patients. Multiple oncology studies are now exploring the utility of IL-15 superagonists and heterodimers to expand both CD8^+^ T cells and NK cells and recent studies evaluated these molecules in the SIV macaque model (140–143). Additional cytokine therapeutics (i.e., IL-21 and IFN-α) could also be attractive targets to mimic or induce the conditions in natural hosts that are conducive to virus clearance in the LNs. Recently, the use of NKG2A inhibitors has also been suggested as an attractive approach in HIV cure strategies (144). Many open questions remain, including delineation of factors responsible for the high IL-15 production in LN follicles, the maintenance of an intact FDC network, the upregulation of CXCR5 on NK cells in LN and the very rapid innate antiviral responses in natural hosts. The remaining gaps in the knowledge base will require future studies to understand how natural hosts reduce inflammation and how they protect LN architecture. Such ongoing studies are hoped to direct future strategies aimed at granting permissive entry of relevant effector cells into the highly restricted lymphoid follicles, thus creating a unique opportunity for reservoir clearance and representing a further step toward HIV remission and cure. Altogether, studies in natural hosts of SIV continue to reveal clues highly relevant for understanding and managing HIV infection in humans.

## Author Contributions

NH, SB, MP, RR, and MM-T wrote the review. NH designed the figure and the table. RR and MM-T edited the text. MM-T composed and oversaw the chapters.

## Conflict of Interest Statement

The authors declare that the research was conducted in the absence of any commercial or financial relationships that could be construed as a potential conflict of interest. The handling Editor declared a shared affiliation, though no other collaboration, with several of the authors SB, MP.
